# eVLP compound delivery breaks the prime editing efficiency ceiling

**DOI:** 10.1016/j.xgen.2026.101333

**Published:** 2026-08-12

**Authors:** Margaret R. Wang, Francisco J. Sánchez-Rivera

**Affiliations:** 1 Department of Biology, Massachusetts Institute of Technology, Cambridge, MA 02142, USA; 2 David H. Koch Institute for Integrative Cancer Research, Massachusetts Institute of Technology, Cambridge, MA 02142, USA; 3 MIT Center for Precision Cancer Medicine, Massachusetts Institute of Technology, Cambridge, MA 02139, USA

## Abstract

Prime editing could resolve gene variant function at scale, but the editing machinery needs to be delivered efficiently and reproducibly. Langley, Baudrier, et al.^1^ show that timing of editor delivery is rate limiting and introduce PRIME-VLP, a repeated dosing strategy with engineered virus-like particles that improves editing efficiency and screening performance.

## Teaser

Prime editing could resolve gene variant function at scale, but the editing machinery needs to be delivered efficiently and reproducibly. Langley, Baudrier, et al. show that timing of editor delivery is rate limiting and introduce PRIME-VLP, a repeated dosing strategy with engineered virus-like particles that improves editing efficiency and screening performance.

## Main text

Prime editing is a precision genome editing technology that enables installation of small edits in a specified location without introducing toxic double-stranded breaks.^2^ Prime editing tools could functionally sort millions of human genetic variants into those that are benign or that drive disease, which has direct implications for disease diagnosis, prognosis, and precision medicine applications. Scaling prime editing into a high-throughput screening format can map thousands of genetic variants to their functional consequences in a single experiment.^3^ This has proven difficult, in large part due to delivery challenges: prime editors are large, multidomain protein and RNA machines that are difficult to efficiently introduce into cells and maximize editing consistently enough to generate robust screen results.

**Figure 1:**
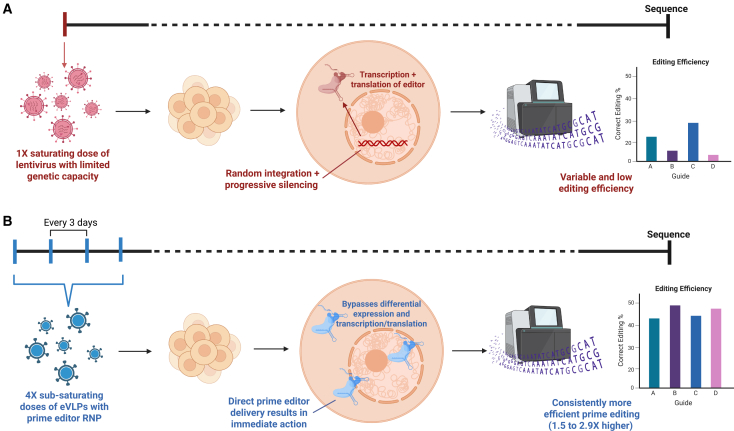
Overcoming delivery bottlenecks to improve prime editing efficiency and enable robust large-scale screens (A) Conventional lentiviral delivery is constrained by the limited cargo capacity of lentivirus and by random integration and progressive silencing of the transgene encoding the guide and editor, as well as delayed editing due to the need for expression and assembly of the prime editing complex. (B) Langley, Baudrier, et al.^1^ overcome this limitation by using eVLPs that directly deliver pre-assembled prime editor ribonucleoprotein (RNP) complexes in four sub-saturating doses at 3-day intervals. Each dose acts immediately by skipping assembly steps and circumventing random integration and progressive silencing, achieving a 1.5- to 2.9-fold higher editing efficiency across diverse genomic targets and human cell types. Biorender was used to create this figure.

Current prime editing screens rely on lentiviral vectors to deliver prime editors into cells (Figure 1A). Lentiviruses have limited cargo capacity,^4^ making it difficult to package the large prime editor protein complex. Once a transgene integrates into the genome, prime editor expression becomes mosaic and is progressively silenced.^5^ To address this, Langley, Baudrier, et al.^1^ introduce PRIME-VLP, a compound delivery method using engineered virus-like particles (eVLPs) that substantially improves the capacity for large-scale functional screening of genetic variants (Figure 1B).

Unlike lentiviruses, eVLPs do not integrate the editor into the host genome. Instead, they deliver pre-assembled editor protein complexes directly into the cell, immediately triggering editing that gradually subsides as the complexes naturally degrade.^6^ Because this editing window is transient, the authors mapped the kinetics of eVLP-mediated editing and found that it increased rapidly over the first 2 days before plateauing by day 3, by which point >90% of edits had already accumulated. This suggests that pre-assembled complexes act within hours and are likely cleared quickly, such that a single dose is largely spent before editing is complete. Loading more editor into a single dose produced diminishing returns beyond a certain threshold; a sub-saturating dose achieved nearly 93% of the editing generated by a four-times-higher dose. Instead of delivering a single large, oversaturating dose of the prime editor, PRIME-VLP is administered as four controlled, sub-saturating doses spaced 3 days apart, which substantially increase editing efficiency (Figure 1B). Because eVLPs lack an integrating viral genome, they do not trigger the antiviral defense response in cells.

The authors compared PRIME-VLP performance against a single saturating dose of eVLPs across six endogenous genomic targets and four human cell lines. PRIME-VLP consistently outperformed single dosing, improving editing efficiency from 1.5- to 2.9-fold. Importantly, the performance of PRIME-VLP extended across editor architectures, from cytosine and adenine base editors to next-generation prime editors like PE7. PRIME-VLP also improved base editing in primary human T cells, which are difficult to engineer. Critically, there was no detectable increase in off-target editing or transcriptional changes, and cell viability was preserved. A single high eVLP dose provoked a stronger transcriptional response compared to PRIME-VLP, and both resolved to baseline over time.

The authors then adapted PRIME-VLP to high-throughput screening by decoupling the editor from the guide library. The prime editor protein is delivered by eVLP, while a collection of prime editing guide RNAs (pegRNAs) specifying each edit and genomic location are delivered separately by lentivirus. This rational division of labor was effective because pegRNAs can be readily packaged into lentiviruses and integrated into target cells while the prime editor protein is supplied transiently with eVLPs, avoiding editor silencing. A 6,000-pegRNA benchmark screen targeting *TP53* in HEK293T cells achieved a 2.8-fold higher mean prime editing efficiency and increased the fraction of pegRNAs with active editing from 44.2% to 82.1%. PRIME-VLP screens were also reproducible, with pegRNA editing rates correlating across replicates at R2 = 0.82 for eVLP versus R2 = 0.2 for prime editor lentivirus. Lentiviral editor expression decreased by 74%–82% over time, whereas eVLP-mediated editing continued to accumulate over multiple rounds of delivery.

A549 human lung adenocarcinoma cells carry two normal copies of *TP53*, and cells harboring *TP53* mutations that disable p53 function can be selected in culture with Nutlin-3. This drug blocks the interaction between the negative regulator MDM2 and p53, leading to p53 stabilization and cell-cycle arrest in cells with intact p53 signaling. A PRIME-VLP screen not only recovered known loss-of-function and dominant negative *TP53* variants, including hotspot mutations at residue R248, but also validated five previously uncharacterized variants, including V173G and W146G. Most mutations were independently supported by their recurrence in human tumor sequencing cohorts, indicating that PRIME-VLP can be used to discover and functionally characterize functional genetic variants.

This study marks a notable advance in precision genome editing, with implications for biological questions that can be addressed with scalable genetic perturbations.^7^^,^^8^ By defining the kinetics of eVLP delivery of prime editor ribonucleotide proteins and adding a compound delivery strategy, Langley, Baudrier, et al.^1^ demonstrate that PRIME-VLP outperforms the current lentiviral gold standard for large-scale screening of genetic variants.

Open questions remain, however. PRIME-VLP still shows significant editing variability across cell types, likely reflecting differences in cellular uptake of eVLPs. It will thus be important to test PRIME-VLP across a range of primary and clinically relevant cells and organoids. The selection window may also need to be extended beyond the 11-day Nutlin-3 selection window used for *TP53* mutations*,* which was shorter than similar screens.^3^ The exceptional performance of PRIME-VLP indicates that realizing the promise of prime editing relies both on advances to improve prime editors themselves and alternative delivery methods. Because eVLPs show promise for therapeutic RNP delivery *in vivo*, a strategy that also maximizes their editing efficiency narrows the distance between functional screening and clinical translation. As prime editing technology matures, pairing editing with efficient, reproducible, transgene-free delivery will be essential for obtaining biological and clinical insight with functional genomic screens.

## Acknowledgments

Work in the Sánchez-Rivera laboratory is supported by the Howard Hughes Medical Institute (Hanna Gray Fellowship, GT15656), the V Foundation for Cancer Research (V2022–028), the National Cancer Institute (Cancer Center Support Grant P30-CA014051, NCI 1P01CA291694–01A1), the Virginia and D.K. Ludwig Fund for Cancer Research, the MIT Center for Precision Cancer Medicine (CPCM), the MIT HEALS Initiative, the Koch Institute Frontier Research Program, the Casey and Family Foundation Cancer Research Fund, Michael (1957) and Inara Erdei Fund, the MIT Research Support Committee, the Upstage Lung Cancer Foundation, and a Traditional Project Award from the Bridge Project, a partnership between the Koch Institute for Integrative Cancer Research at MIT and the Dana-Farber/Harvard Cancer Center. M.R.W. is also supported by NIH T32GM136540. Figure 1 was created in BioRender.

## Declaration of interests

F.J.S.R. has consulted for Repare Therapeutics, Ono Pharma, and Merck.
